# Abrasion-Resistant Layered Superhydrophobic Coatings: Fabrication, Performance Evaluation, and Mechanistic Analysis of Ice Adhesion

**DOI:** 10.3390/polym18091077

**Published:** 2026-04-29

**Authors:** Gaoquan Li, Lee Li, Biao Huang, Kang Luo, Yi Xie, Tao Xu, Wenhua Wu

**Affiliations:** 1 School of Electrical and Electronic Engineering, Huazhong University of Science and Technology, Wuhan 430074, China; 2 State Key Laboratory of Silicate Materials for Architectures, Wuhan University of Technology, Wuhan 430070, China; 3 China Electric Power Research Institute, Wuhan 430074, China

**Keywords:** layered superhydrophobic coatings, abrasion resistance, ice adhesion strength, ice adhesion mechanism, electrical insulation

## Abstract

Superhydrophobic coatings are regarded as a promising passive anti-icing strategy; however, their practical engineering application, particularly in electrical insulation, is severely hindered by the performance deterioration caused by mechanical damage and a lack of theoretical understanding of microscopic ice adhesion mechanisms. In this study, a layered polymer composite coating was designed to resolve the trade-off between abrasion resistance and low ice adhesion. The chemistry of the coating relies on a synergistic “primer–topcoat” design: the primer consists of an epoxy resin matrix chemically modified by amino silicone oil to lower its surface energy and improve toughness, while the topcoat features hierarchical SiO_2_ clusters functionalized with hexamethyldisilazane (HMDS) and silane coupling agents. This architecture was fabricated via a controllable layer-by-layer spraying method. Systematic investigations revealed that the hierarchical micro/nanostructure, composed of microscale protrusions and nanoscale SiO_2_ clusters, provides excellent superhydrophobicity (contact angle of 155.2°, sliding angle of 2°). Crucially, the crosslinked polymer network and stable siloxane (Si-O-Si) covalent bonding ensure that the coating maintains its functionality after a cumulative sand impact of 3 kg, demonstrating superior mechanical durability. Furthermore, differentiated theoretical models for ice adhesion in Cassie–Baxter and Wenzel states were established based on intermolecular interactions, identifying that maintaining a stable Cassie–Baxter state is key to reducing adhesion. This study offers a robust approach to balancing functionality and durability in polymer composites through synergistic structural design, providing both a scalable fabrication strategy and a quantitative theoretical framework for understanding interfacial ice adhesion.

## 1. Introduction

Ice accretion is widespread in service environments such as aviation, wind turbine blades, power transmission lines, and railways, leading to increased structural loads, deteriorated aerodynamic performance, and significant risks to operational safety [1,2,3]. Specifically, icing on power transmission lines can reduce the insulating performance of insulators and increase mechanical loads on towers, potentially causing electrical faults like ice flashover or structural accidents such as tower collapse [4,5]. Likewise, non-uniform ice on wind turbine blades aggravates vibration and noise while reducing power generation efficiency [6,7]. Consequently, developing efficient and stable anti-icing technologies is of great significance. Inspired by natural superhydrophobic interfaces such as lotus leaves, superhydrophobic coatings utilize low surface energy and micro/nanoscale rough structures to delay the icing process by increasing the apparent contact angle and reducing the actual solid–liquid contact area [8,9]. The synergistic effect of these features enables droplets to preferentially maintain a stable Cassie–Baxter state during freezing [10], where a trapped air layer within the microstructures hinders the inward migration of the three-phase contact line, significantly reducing the actual contact area and lowering the ice adhesion strength [11].

In recent years, research on reducing ice adhesion strength using superhydrophobic coatings has shifted from the sole pursuit of low ice adhesion to an integrated design that also takes into account durability, environmental adaptability, and engineering feasibility. Cao et al. [12] fabricated an F-SiO_2_ superhydrophobic anti-icing coating on a metal surface via a template method, which reduced the ice adhesion strength to 14.8 kPa, representing a decrease of approximately 90% compared with that of the bare substrate. Moreover, the coating still maintained good anti-icing performance after 150 icing/deicing cycles, indicating that a stable micro/nanostructure with low surface energy can effectively reduce the interfacial adhesion between ice and the substrate. In terms of material resilience, Lei et al. [13] incorporated modified nanoscale SiO_2_ into a polyurethane system to prepare a superhydrophobic coating with both abrasion resistance and impact resistance. At −15 °C, the coating prolonged the droplet freezing time to 700 s and kept the ice adhesion strength below 13 kPa even after 25 freeze–thaw cycles. Furthermore, to address environmental complexity, Mao et al. [14] further combined the photothermal effect with a superhydrophobic structure, thereby delaying icing under low-temperature, high-humidity, and low-light conditions and reducing the ice adhesion strength on the aluminum alloy surface from 205.3 kPa to 36.3 kPa, indicating that the introduction of photothermal functionality helps improve anti-icing performance in complex environments. For engineering applications, Xu et al. [15] proposed a bilayer synergistic strategy, which reduced the ice adhesion strength to 12.2 kPa while still maintaining good hydrophobicity and anti-icing performance after mechanical durability tests. These diverse strategies collectively suggest that the synergistic regulation of interfacial structure and surface chemistry can effectively enhance anti-icing capability under complex operating conditions.

Although relevant studies have made progress in reducing ice adhesion strength and improving service performance, a comprehensive understanding of the microscopic ice adhesion mechanism on superhydrophobic surfaces remains elusive due to the complex multi-physics coupling of droplet impact, spreading, condensation, freezing, and interfacial failure. Existing studies have mostly investigated ice adhesion strength by isolating specific influencing factors through empirical research. Wang et al. [16] found that variations in the nanoparticle content altered the surface roughness features of a superhydrophobic composite coating, causing the ice adhesion strength to first decrease and then gradually stabilize, which indicates that surface topography plays an important role in the ice–solid interfacial interaction. Similarly, Misiiuk et al. [17] pointed out that the direction, shape, and scale of topology-induced wettability gradients all affect ice adhesion strength, and that enhanced hydrophobicity does not necessarily correspond to lower ice adhesion strength. The ice removal direction can likewise influence the measured results. In addition to surface structure, environmental factors can also significantly affect ice adhesion strength. Fu et al. [18] found that an increase in ambient humidity significantly increased the ice adhesion strength, because humid air more readily condensed within the microstructures and enhanced interfacial adhesion. Chatterjee et al. [19] showed that impurities such as salts, surfactants, and solvents, as well as variations in freezing temperature, can all significantly affect the interfacial adhesion behavior of impure ice. Furthermore, Lv et al. [20] found that a decrease in temperature significantly increased the ice adhesion strength, while surface roughness aggravated adhesion by enhancing mechanical interlocking; in contrast, functional anti-icing coatings could effectively reduce such interfacial adhesion. While these studies provide valuable insights into specific environmental and structural parameters, they often focus on macroscopic observations rather than the underlying microscopic force interactions.

Overall, although macroscopic characterization of ice adhesion has been widely reported, the microscopic force interactions governing ice adhesion under different wetting states remain insufficiently understood. For the protection of electrical equipment, coatings are required to simultaneously achieve effective anti-icing performance and satisfactory mechanical durability, yet the interfacial adhesion mechanisms associated with different contact states have not been fully clarified. In this study, a superhydrophobic coating with a layered spraying architecture was developed to investigate ice adhesion by combining a microscopic theoretical model with experimental characterization. A model based on intermolecular interactions was established, and ice adhesion strength tests were conducted to analyze the force mechanisms in the Cassie–Baxter and Wenzel states. The results clarify the relationship between surface structure and ice adhesion, and provide theoretical support for the design of durable insulating surfaces for power system components operating under icing conditions.

## 2. Sample Preparation and Performance Testing

### 2.1. Sample Preparation

To clarify the experimental conditions for the fabrication and performance testing of the superhydrophobic coating, Table 1 lists the names and specifications of the raw materials used for coating preparation, while Table 2 presents the names and models of the equipment employed during the fabrication and testing processes.

#### 2.1.1. Preparation of the Primer and Topcoat

To meet different environmental conditions and application requirements, the superhydrophobic coating prepared in this study consisted of a primer and a topcoat. The primer should possess good adhesion so that it can firmly bond to the substrate surface. Epoxy resin is widely used as an additive or binder in composite materials because of its good abrasion resistance and adhesion [21,22]. However, such material systems often contain a large number of polar groups, such as epoxy groups, ether bonds, and hydroxyl groups, which impart relatively high hydrophilicity and wettability. Therefore, modification of the epoxy resin is necessary. As an organosilicon material, amino silicone oil has a relatively low binding energy with water molecules. Modifying the epoxy resin with amino silicone oil can effectively improve the mechanical properties and hydrophobicity of the primer matrix material.

**Figure 1:**
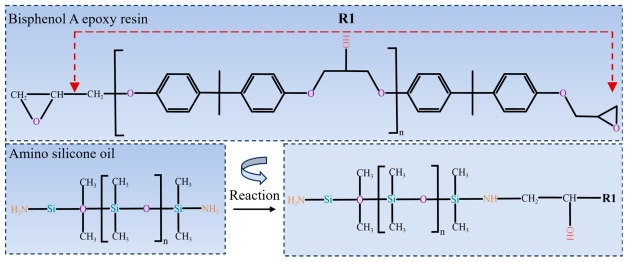
Reaction mechanism of bisphenol A epoxy resin and amino silicone oil. The red dashed arrows define the chemical moiety represented by R1.

The specific procedure for preparing the primer material through the copolymerization of epoxy resin and amino silicone oil with a mass fraction of 10% was as follows: bisphenol A epoxy resin and amino silicone oil were dissolved in acetone at a mass ratio of 6:1, and the mixture was then thoroughly stirred with a magnetic stirrer at 70 °C and 200 rpm for 3 h; After the reaction was completed and the mixture had cooled to room temperature, 1 g of nylon powder with a certain mass fraction was added and magnetically stirred for 2 h to ensure uniform dispersion in the solution. Subsequently, the curing agent 4,4′-diaminodiphenylmethane (DDM) was introduced at a ratio of *n* (epoxy group): *n* (amino hydrogen) = 1:1, and the mixture was further stirred thoroughly for 2 h to obtain the modified epoxy resin primer solution. The preparation principle is illustrated in Figure 1. Because the molecular structure of amino silicone oil contains both siloxane chains and amino groups, on the one hand, the active hydrogen atoms in the amino groups can react with the epoxy groups in the epoxy resin, thereby promoting epoxy ring opening and molecular crosslinking to form a more stable cured network [23], which improves the abrasion resistance and chemical stability of the epoxy resin. On the other hand, siloxane chains possess good flexibility and low surface tension. After being introduced into the epoxy resin, they can reduce the hydrophilicity of the resin surface and thereby improve its compatibility with low surface energy particles.

**Figure 2:**
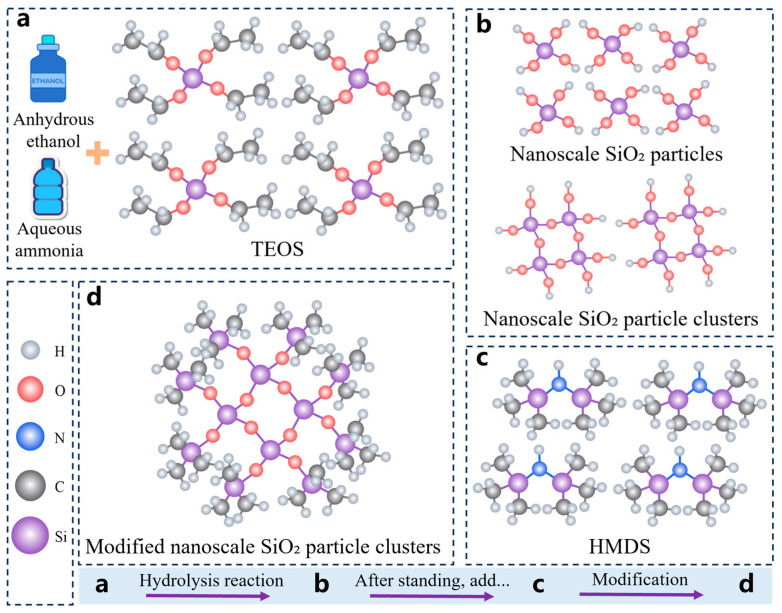
Preparation process of the topcoat material for the superhydrophobic coating. (**a**) Hydrolysis reaction in an alkaline environment; (**b**) nanoscale SiO_2_ particles and particle clusters; (**c**,**d**) surface modification of the SiO_2_ particle clusters.

The topcoat was prepared by a hydrolysis method and mainly consisted of low surface energy substances. Superhydrophobicity was achieved by constructing a micro/nanoscale rough structure, and the specific preparation procedure is shown in Figure 2.

10 mL of aqueous ammonia was added to 100 mL of anhydrous ethanol (anhydrous ethanol: aqueous ammonia = 10:1), and the mixture was thoroughly stirred at room temperature to establish an alkaline environment. After a period of stirring, 30 mL of the precursor tetraethoxysilane (TEOS) was slowly added to the solution, allowing it to undergo hydrolysis in the alkaline environment to form nanoscale SiO_2_ particles. During this process, the hydrolyzed nanoscale SiO_2_ particles further underwent condensation reactions to form SiO_2_ particle clusters. After standing at room temperature for 12 h, 55 mL of hexamethyldisilazane (HMDS) and the silane coupling agent KH560 were slowly added to the dispersion of SiO_2_ particle clusters. During the hydrolysis process, HMDS undergoes chemical reactions in which the silanol groups (Si−OH) at its hydrophilic end condense with the hydroxyl groups (−OH) on the surface of the nanoscale SiO_2_ particles to form stable siloxane bonds (Si−O−Si). Meanwhile, the methyl groups (−CH_3_) at its hydrophobic end are oriented outward, thereby changing the surface chemical properties of the SiO_2_ particles. As a result, the originally hydrophilic hydroxyl groups are replaced by hydrophobic methyl groups, transforming the SiO_2_ particle surface from hydrophilic to hydrophobic and ultimately yielding the topcoat material for the superhydrophobic coating.

#### 2.1.2. Coating Preparation Method

**Figure 3:**
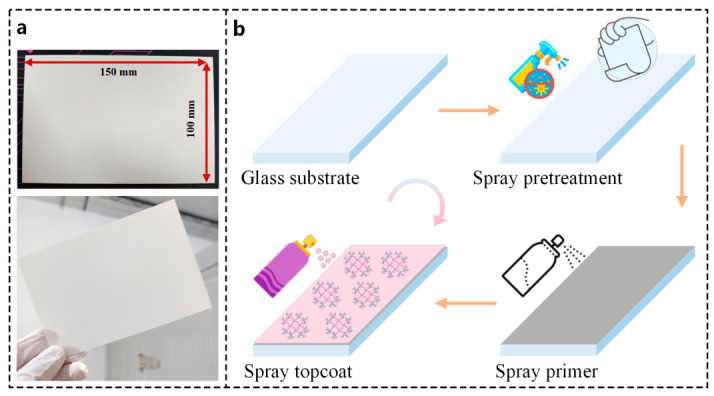
Preparation process of the superhydrophobic coating. (**a**) Prepared superhydrophobic coating; (**b**) schematic illustration of the preparation process of the superhydrophobic coating.

Glass substrates with dimensions of 150 mm × 100 mm were used in this study. Before spraying, the substrate surface was first cleaned with anhydrous ethanol to remove surface impurities to ensure optimal interfacial bonding, and was then roughened with sandpaper to increase the specific surface area and promote mechanical interlocking between the substrate and the primer. The preparation procedure of the superhydrophobic coating is shown in Figure 3b.

First, the prepared primer material was sprayed onto the treated substrate surface to serve as a robust adhesive transition layer. After 3~5 spray cycles, the substrate was placed in a forced-air drying oven at 50 °C for 2 h to allow complete solvent evaporation and provide a stable foundation for the topcoat application. Then, using the same spraying method, the topcoat material was sprayed onto the cured primer surface. The spraying speed of the topcoat was strictly controlled, and after 2~4 spray cycles, the substrate was left to cure at room temperature for 24 h, allowing the coating structure to fully stabilize, yielding the complete superhydrophobic coating, as shown in Figure 3a. The degree of roughness of the micro/nanostructure on the coating surface can be controlled through the preparation method and fabrication process. Adjusting the spraying distance and speed can effectively alter the coating thickness and dispersion, thereby modulating the surface topography and ultimately determining the final wetting properties.

### 2.2. Test Methods and Results Analysis

#### 2.2.1. Microstructural Characteristics of the Coating Surface

**Figure 4:**
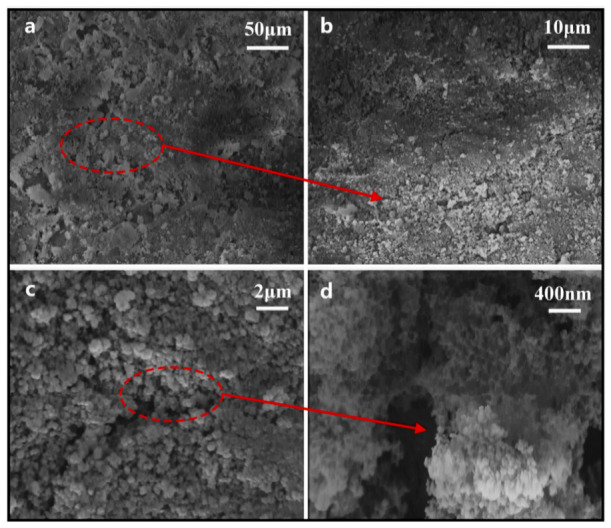
Surface morphology of the superhydrophobic coating. (**a**) 50 μm scale; (**b**) 10 μm scale; (**c**) 2 μm scale; (**d**) 400 nm scale.

The surface microstructure of the samples was observed using a Nova field emission scanning electron microscope (FE-SEM). Before testing, the coating material was cut into 1 cm × 1 cm specimens using a glass cutter, and the specimens were fixed onto the sample stage with conductive adhesive tape. To ensure complete drying, the specimens were treated under vacuum conditions. Subsequently, a high-resolution sputter coater was used to uniformly coat the sample surface with a thin gold layer to enhance conductivity and improve imaging clarity [24]. The microscale and nanoscale surface morphology of the coating is shown in Figure 4.

As shown in Figure 4a, uniformly distributed and continuous microscale protrusions were formed on the coating surface, indicating good surface coverage over a relatively large scale range. As shown in Figure 4b, the surface further exhibited a relatively regular three-dimensional array of protrusions, showing a pronounced microscale undulating morphology. With increasing magnification, Figure 4c shows that smaller-scale roughness units were still distributed on the surface of the protrusion-like microstructures, indicating that the surface possessed multiscale structural characteristics. Further, as shown in Figure 4d, the surface nanostructure was mainly composed of accumulated SiO_2_ particle clusters. These particles were interconnected with one another, forming a relatively dense, nanoscale, rough layer. The microscale structures and nanoscale clusters together constructed a typical hierarchical micro/nanoscale rough surface, which not only increased the surface roughness and specific surface area, but also facilitated the distribution and stable presence of low surface energy groups on the surface. The synergistic effect of these two factors effectively reduced the contact and adhesion between droplets and the solid surface, making the droplets more likely to maintain a spherical shape and roll off, thereby endowing the coating with excellent superhydrophobicity.

**Figure 5:**
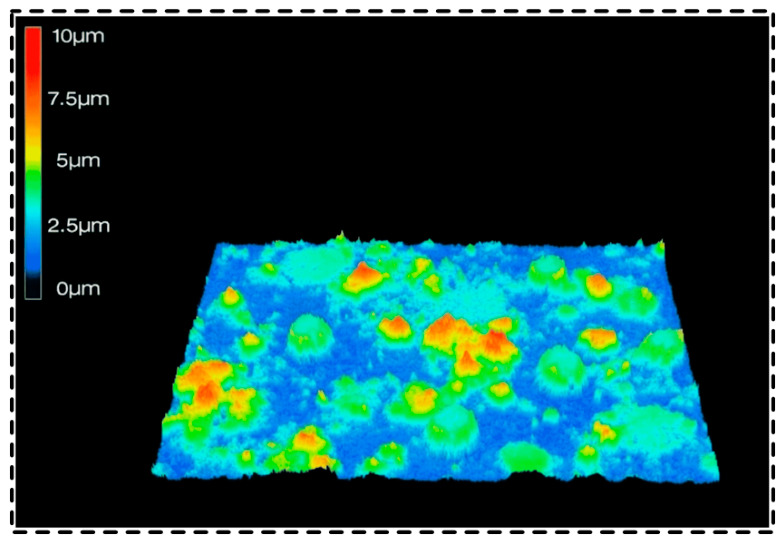
Three-dimensional surface morphology obtained by confocal microscopy.

A confocal microscope was used to perform laser scanning characterization of the superhydrophobic coating surface, and its three-dimensional surface morphology was obtained, as shown in Figure 5. The three-dimensional morphology provides a more intuitive visualization of the undulating features and spatial distribution of the coating surface, offering an important basis for analyzing its surface microstructure. Compared with two-dimensional morphological observation, three-dimensional characterization can more comprehensively reveal the roughening characteristics of the coating surface and the manner in which they are constructed, thereby helping to further understand the intrinsic relationship between surface morphology and wettability.

The surface of the superhydrophobic coating exhibited pronounced undulating features, with unevenly distributed rough structures and certain variations in height, resulting in relatively significant fluctuations in the overall surface morphology. The profile analysis results showed that the average height of the surface profile peaks was approximately 4 μm, indicating that the coating possessed a relatively pronounced microstructural roughness. Such an undulating surface morphology helps enhance the roughness of the surface and provides favorable conditions for air entrapment at the solid–liquid interface, thereby exerting an important influence on the wetting behavior of the surface. The surface roughness $R_{q}$ of the coating was calculated according to Equation (1):

(1) $$R_{q}=\sqrt{\sum_{i=1}^{n}{\left(H_{i}-\bar{H}\right)}^{2}/n}$$

where $H_{i}$ is the height of each surface profile peak; $\bar{H}$ is the average height of the surface profile peaks; and n is the number of profile peaks within the measured area.

#### 2.2.2. Surface Elemental Composition Analysis

**Figure 6:**
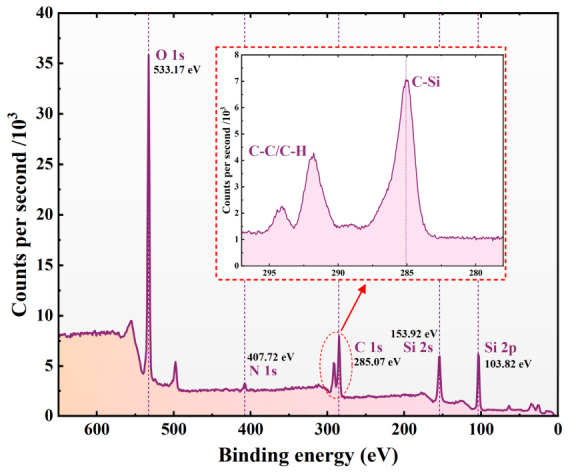
XPS wide-scan spectrum of the sample surface elements.

To characterize the elemental composition of the superhydrophobic coating surface, the surface was analyzed using an X-ray photoelectron spectrometer (XPS), and the wide-scan spectrum of the surface elements was obtained. Before testing, the coating material was cut into 1 cm × 1 cm specimens using a glass cutter, and the specimens were then ultrasonically cleaned successively in acetone and anhydrous ethanol three times for each solvent, with each cleaning step lasting 15 min. After cleaning, the specimens were degassed for 12 h to remove surface-adsorbed impurities. The wide-scan results can be used to preliminarily identify the main elements on the coating surface, providing a basis for further analysis of its surface chemical composition, as shown in Figure 6.

The wide-scan spectrum shows that four main elements, namely C, N, O, and Si, were detected on the coating surface. Their corresponding characteristic binding energy peaks appeared at 285.07 eV (C 1s), 407.72 eV (N 1s), 533.17 eV (O 1s), as well as 153.92 eV (Si 2s) and 103.82 eV (Si 2p), respectively. According to the relative intensities of the peaks, the peak areas of C 1s, O 1s, Si 2s, and Si 2p were relatively large, indicating that C, O, and Si were the main constituent elements on the coating surface, whereas the N content was relatively low.

Further analysis showed that the Si and O signals mainly originated from the SiO_2_ components on the surface, indicating that the inorganic silicon–oxygen structure was dominant in the near-surface region of the coating; The relatively strong C 1s signal further indicates that the coating surface also contained a considerable amount of organic components, which is consistent with the presence of the organic resin matrix and the surface hydrophobic modification layer. The appearance of the N 1s peak indicates that nitrogen-containing chemical groups were introduced onto the coating surface, providing chemical evidence for the construction of surface functional components. Notably, the C 1s region exhibited a continuous peak profile. Combined with the high-resolution spectral analysis, this feature was found to mainly originate from C–Si bonds and C–C/C–H bonds. The presence of C–Si bonds indicates the formation of effective chemical bonding between the inorganic SiO_2_ component and the organic phase, whereas the C–C/C–H bonds mainly arise from the resin matrix or the organic modification chains on the surface.

Overall, the XPS results indicate that inorganic silicon–oxygen structures and organic functional components coexist synergistically on the coating surface.

#### 2.2.3. Surface Wettability Characterization

**Figure 7:**
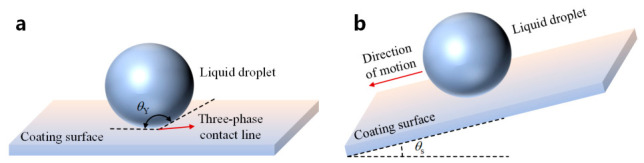
Schematic illustration of the measurements of static contact angle and sliding angle. (**a**) Static contact angle; (**b**) sliding angle.

**Figure 8:**
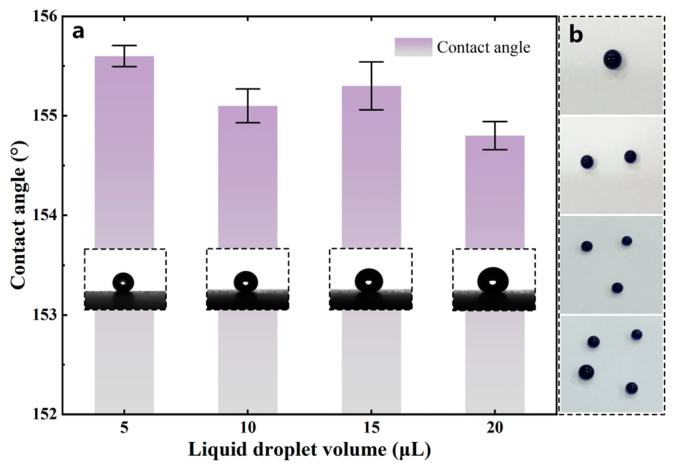
Variation in static contact angle with droplet volume. (**a**) Static contact angle test results for droplets with different volumes; (**b**) wetting behavior of methylene blue aqueous solution droplets with different volumes on the superhydrophobic coating.

The wettability of the material was characterized by the static contact angle and sliding angle. The static contact angle $\theta_{Y}$ is defined as the angle between the tangent to the liquid–gas interface and the solid surface at the three-phase contact line, while the sliding angle $\theta_{s}$ is defined as the critical inclination angle at which a droplet begins to roll off on an inclined solid surface, as shown in Figure 7. For the static contact angle measurement, a KZS-30 contact angle meter was used. Deionized water droplets with different volumes [25], namely 5, 10, 15, and 20 μL, were deposited onto the coating surface using a microsyringe. Ten measurements were conducted for each droplet volume, and the averaged results are shown in Figure 8a.

Within the allowable range of experimental error, the average contact angle error for droplets of the four tested volumes was less than 1°, and the contact angles for all droplet volumes were higher than 154°, with an average contact angle of 155.2°. The results indicate that droplet volume has little influence on the measured contact angle of the superhydrophobic coating, demonstrating that the coating possesses good static wettability stability. The wetting behavior of methylene blue aqueous solution droplets with different volumes on the superhydrophobic coating is shown in Figure 8b. It can be seen that, within the tested droplet volume range, the methylene blue aqueous solution remained in a relatively stable spherical state on the coating surface, without obvious spreading or penetration, indicating that the coating possesses excellent resistance to wetting. As the droplet volume increased, the overall droplet contour remained relatively intact; the minor deformation caused by gravity was effectively resisted by the stable Cassie–Baxter state. Additionally, larger droplets facilitate sliding as their increased mass provides sufficient driving force to overcome interfacial adhesion, indicating that the superhydrophobic coating surface possessed low surface energy and exhibited good liquid repellency.

**Figure 9:**
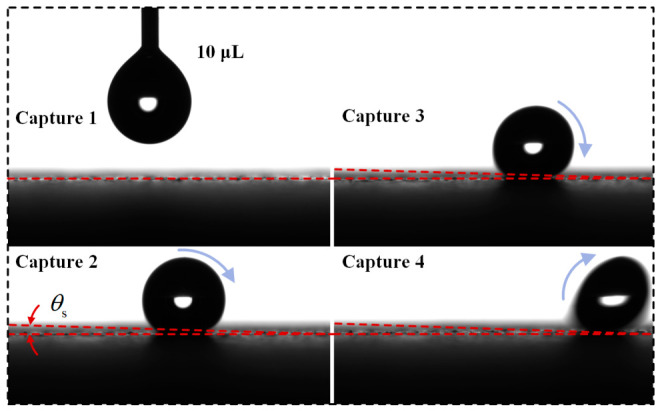
Sliding angle measurement process.

**Figure 10:**
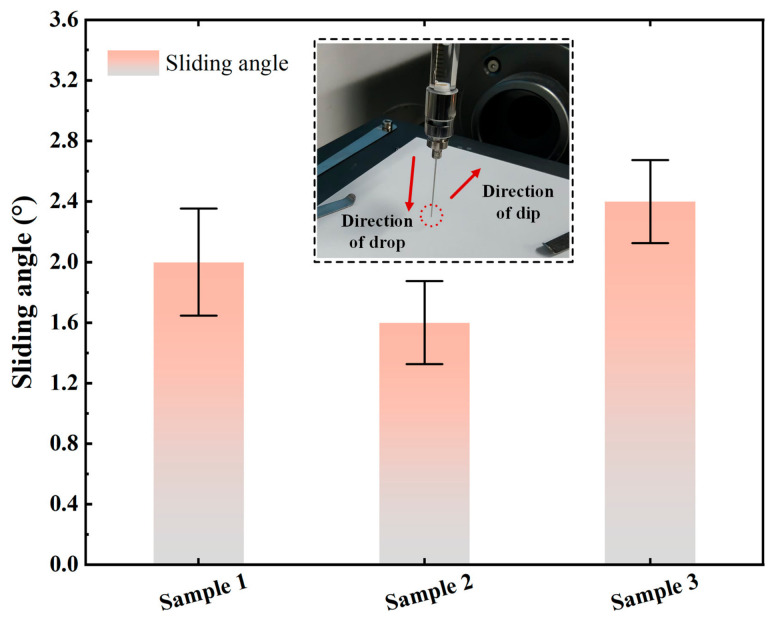
Sliding angle measurement results.

For the sliding angle measurement, three groups of coating samples were selected, and the volume of the deionized water droplet was set at 10 μL. The samples were tilted at a constant rate of 1°/s using a sliding angle measuring device, and the moment at which the droplet began to roll on the coating surface was captured [25]. The scale reading of the device at that moment was recorded, and the measurement process is shown in Figure 9. For each group of samples, the sliding angle was measured at five different positions to obtain reliable dynamic wettability data, and the averaged results are shown in Figure 10. The sliding angles of all three groups of samples were lower than 4°, with an average sliding angle of 2°. During the test, once the measuring platform was slightly tilted, the droplet rapidly rolled off the surface under gravity, indicating that the coating exerted little resistance to droplet motion and had weak surface adhesion. Therefore, it exhibited good dynamic wettability and self-cleaning capability.

#### 2.2.4. Abrasion Resistance Test

**Figure 11:**
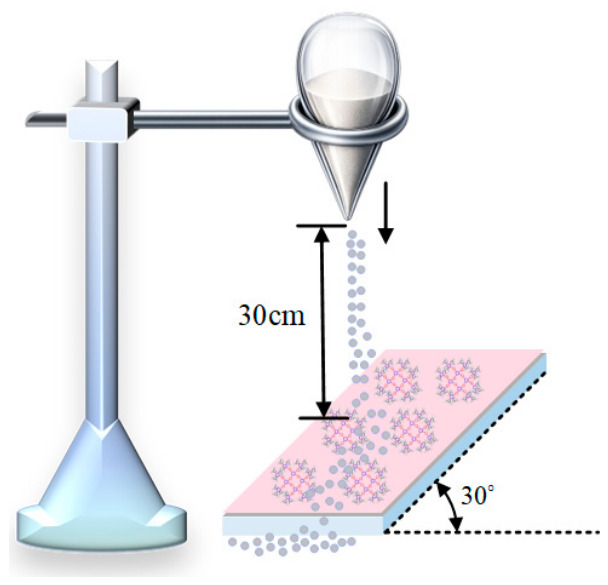
Schematic illustration of the sand impact test.

**Figure 12:**
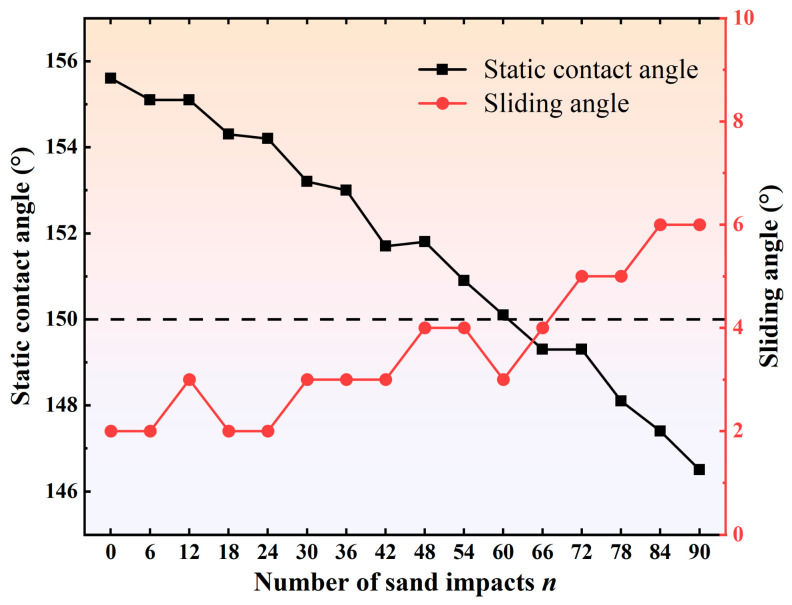
Variations in static contact angle and sliding angle after sand impact.

During practical use, superhydrophobic coatings are subjected to various complex environmental conditions and inevitably experience different degrees of abrasion. Therefore, their abrasion resistance has become a key evaluation criterion. In this study, a sand impact test, which is more representative of conditions commonly encountered in natural environments, was adopted to quantify the abrasion resistance of the prepared superhydrophobic coating surface [26]. The schematic illustration of the test is shown in Figure 11. First, the prepared sample was fixed on an inclined plane with an angle of 30°. Fine sand used for a single sand impact test was weighed to 50 g using an electronic balance. The sand outlet of a separatory funnel was positioned vertically above the center of the sample, and fine sand with an average particle diameter of approximately 200 μm was continuously released from a height of 30 cm to impact the sample surface. This procedure was repeated cyclically, and the variations in static contact angle and sliding angle were measured every six cycles, as shown in Figure 12. With an increasing number of sand impacts, the contact angle of the superhydrophobic coating gradually decreased, while the sliding angle remained at or below 6° throughout the test. When the number of impacts reached 66, the static contact angle dropped below 150°, indicating that the coating no longer retained its superhydrophobicity. This is mainly because continuous sand impact damaged the micro/nanoscale rough structures on the coating surface, resulting in the gradual degradation of the surface functionality.

Comparison of the abrasion resistance of this coating with that of superhydrophobic coatings reported in the literature shows that the cumulative sand impact mass tolerated by the reported coatings was generally below 1 kg [27,28], whereas the coating prepared in this study still retained its superhydrophobicity after being subjected to a cumulative sand impact mass of 3 kg, demonstrating good impact-abrasion resistance. The reasons can be analyzed from two aspects. On the one hand, the primer was based on epoxy resin modified with amino silicone oil, and the resulting crosslinked network structure effectively improved the compatibility and bonding strength between the primer and the surface-modified particles; On the other hand, after the SiO_2_ particles were treated with the silane coupling agent KH560, the alkoxy groups at one end of the coupling agent could react with the hydroxyl groups on the SiO_2_ particle surface to form stable siloxane bonds, while the active groups at the other end could undergo ring-opening reactions with the epoxy groups in the amino silicone oil-modified epoxy resin, thereby further forming stable covalent linkages. This chemical bonding effect significantly enhanced the interfacial bonding strength between the modified particles in the topcoat and the resin surface of the primer, thereby endowing the coating with good abrasion resistance.

#### 2.2.5. Ice Adhesion Strength Test

**Figure 13:**
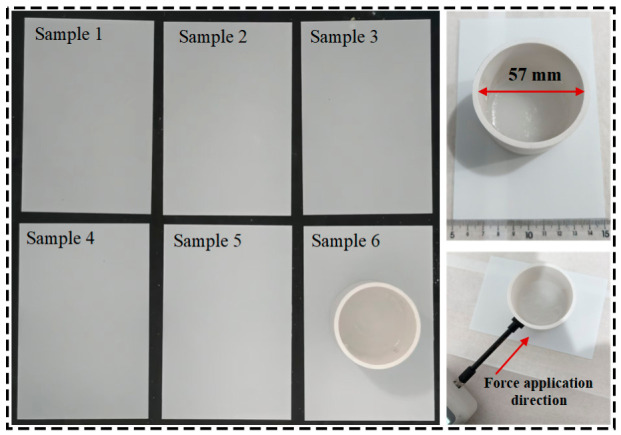
Testing process of ice adhesion strength on superhydrophobic coating samples.

Artificial icing was performed on the superhydrophobic coating surface, using a glass substrate with dimensions of 150 mm × 100 mm. Deionized water was placed in a DW-50 low-temperature test chamber, and the chamber temperature was set to −10 °C. After cooling for 1 h, the coated sample was placed into the chamber. Six identical superhydrophobic coating samples were prepared to reduce experimental error and ensure the statistical stability of the results, and a ring-shaped mold with an inner diameter of 57 mm was placed horizontally on the coating surface, ensuring that there was no gap at the contact interface to facilitate demolding after icing [29], as shown in Figure 13. Then, 5–10 mL of the prepared supercooled deionized water was introduced into the mold, after which the sample was placed in the low-temperature test chamber and frozen for 20 min. The water was allowed to freeze naturally under the effect of surface tension. Once it no longer flowed out from the gap between the coating and the mold, the remaining supercooled deionized water was slowly added. To reduce the influence of the self-weight of the ice on the measurement process, the liquid level height was controlled at 5 mm. The sample was then placed in the low-temperature test chamber and frozen at −15 °C for 2.5 h. After freezing, a force gauge operating in peak mode was immediately used to measure the critical force at which each sample was just pulled off vertically or displaced horizontally. During the experiment, it was ensured that no melting occurred in the ice layer.

**Figure 14:**
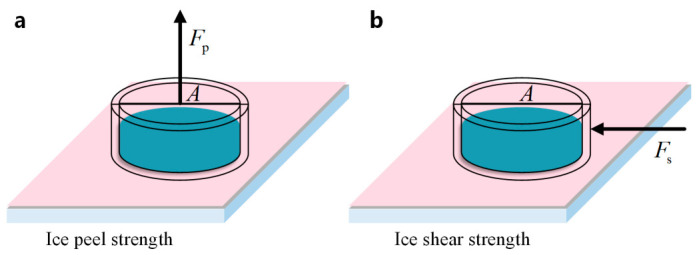
Measurement methods for ice peel strength and ice shear strength. (**a**) Ice peel strength; (**b**) ice shear strength.

**Figure 15:**
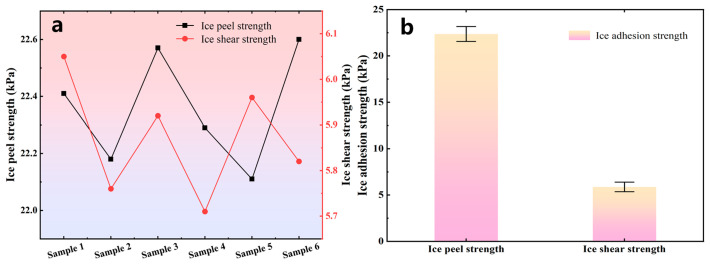
Measurement results of ice peel strength and ice shear strength. (**a**) Measurement results for different samples; (**b**) average values.

The ice peel strength on the sample surface was calculated according to *P*_p_ = *F*_p_/*A*, and the ice shear strength on the sample surface was calculated according to *P*_s_ = *F*_s_/*A*, where *A* is the ice-covered area, as shown in Figure 14a,b. The ice peel strength and ice shear strength of the superhydrophobic coating samples were measured, and the average values were calculated and taken as the test results for the ice peel strength and ice shear strength of the coating, as shown in Figure 15.

The test results showed that the ice peel strength and ice shear strength of the superhydrophobic modified SiO_2_ coating were 22.36 kPa and 5.87 kPa, respectively. In comparison, the ice adhesion strength of the reference RTV coating was found to be significantly higher, far exceeding the values obtained for our superhydrophobic surface [29]. This stark contrast clearly demonstrates the superior anti-icing efficiency conferred by the as-prepared hierarchical structure. The measured data showed little variation, indicating good stability in the ice adhesion behavior of the coating. The low surface energy and micro/nanoscale rough structure of the superhydrophobic coating surface act synergistically to significantly reduce the actual contact area between the ice layer and the coating, thereby weakening the interfacial adhesion. Because the surface does not form a continuous and compact contact with the ice layer, the ice mainly makes localized contact with some of the microscopic protrusions, resulting in a relatively low overall adhesion strength.

Under the same testing conditions, the ice peel strength was significantly higher than the ice shear strength, indicating that the interfacial failure modes of the ice layer were different under normal detachment and tangential sliding conditions. This can be attributed to the fact that under shear loading, the ice layer is more prone to relative sliding along the interface, and the local contact points can fail progressively; therefore, a smaller external force is required to remove the ice. In contrast, under peel loading, the ice layer must detach from the surface as a whole in the normal direction. In addition to the interfacial adhesion, the local anchoring and constraining effects exerted by the microscopic protrusions on the ice layer must also be overcome. Therefore, the peel process is generally more difficult and exhibits a higher peel strength.

## 3. Ice Adhesion Mechanism on the Superhydrophobic Coating Surface

Drawing upon the experimental characterizations in Section 2, this section investigates the ice adhesion mechanism through theoretical modeling. Furthermore, the influence of key parameters on ice adhesion strength is explored to provide a theoretical foundation for strength calculation and coating design optimization.

### 3.1. Theoretical Analysis of Ice Adhesion Strength

The adhesion of ice on superhydrophobic surfaces is governed by the coupled effects of wetting state, icing environment, icing mode, and surface roughness. Its origin is generally attributed to mechanical interlocking induced by the rough surface structures, as well as intermolecular interactions at the interface, such as van der Waals forces and hydrogen bonding [30]. The low surface energy and micro/nanoscale rough structures of the superhydrophobic coating significantly reduce the actual contact area between the ice layer and the coating surface, thereby weakening the van der Waals interactions at the ice–coating interface; Under the typical Cassie–Baxter contact state, the presence of an interfacial air layer suppresses the penetration of ice crystals into the micro/nanostructures, thereby limiting mechanical interlocking. In contrast, the polar functional groups on the coating surface can form hydrogen bonds with water molecules in the ice, thereby enhancing the interaction at the ice–coating interface, and are considered to be a major source of ice adhesion [31,32].

The magnitude of the electrostatic charge $q_{w}$ in water molecules can be determined from the molecular dipole moment and the distance between the opposite charges, as follows:

(2) $$q_{w}=\frac{\sigma_{w}}{\delta }$$

where $\sigma_{w}$ is the dipole moment of water; and δ is the distance between the charges.

According to Coulomb’s law, the electrostatic interaction force $F_{w}$ between water and the coating can be calculated as follows:

(3) $$F_{w}=\frac{q_{w}q_{\mathrm{coating}}}{4\pi \varepsilon_{0}\varepsilon_{w}\delta_{w}^{2}}$$

where $q_{\mathrm{coating}}$ is the electrostatic charge on the coating surface; $\varepsilon_{0}$ is the vacuum permittivity; $\varepsilon_{w}$ is the relative permittivity of water; and $\delta_{w}$ is the equivalent distance between the water molecules and the surface molecules of the coating.

According to the theoretical equation for droplet contact angle proposed by Thomas Young, at the three-phase contact line among air, the water droplet, and the coating surface, the surface tensions at the solid–gas, solid–liquid, and liquid–gas interfaces are in equilibrium:

(4) $$\gamma_{\mathrm{sg}}-\gamma_{\mathrm{sl}}=\gamma_{\mathrm{lg}}\cos\theta_{Y}$$

where $\gamma_{\mathrm{sg}}$ is the surface tension at the solid–gas interface; $\gamma_{\mathrm{sl}}$ is the surface tension at the solid–liquid interface; $\gamma_{\mathrm{lg}}$ is the surface tension at the liquid–gas interface; and $\theta_{Y}$ is the angle between the solid–liquid and liquid–gas interfaces, namely, the static contact angle on the coating surface.

The magnitudes of the surface tensions at the solid–liquid–gas three-phase contact line reflect the interfacial energy per unit area. Accordingly, the work of adhesion per unit area between water and the coating surface, $\omega_{a}$, can be obtained as follows:

(5) $$\omega_{a}=\gamma_{\mathrm{sg}}+\gamma_{\mathrm{lg}}-\gamma_{\mathrm{sl}}$$

By combining Equations (4) and (5), the Young–Dupré equation [33] can be obtained:

(6) $$\omega_{a}=\gamma_{\mathrm{lg}}\left(1+\cos\theta_{Y}\right)$$

The mechanical work $W_{a}$ required to break the equilibrium state of the droplet can be analyzed from the perspective of work:

(7) $$W_{a}=\int_{0}^{\delta_{w}}F_{w}d\delta$$

According to the relationship between the mechanical work required to break the equilibrium state of the droplet and the work of adhesion per unit area between water and the coating surface, the following expression can be obtained:

(8) $$\omega_{a}=\frac{F_{w}\delta_{w}}{2S_{a}}$$

where $S_{a}$ is the contact area between water and the coating surface.

By combining Equations (6) and (8), the adhesion strength $P_{w}$ of water on the coating surface can be obtained as follows:

(9) $$P_{w}=\frac{F_{w}}{S_{a}}=\frac{2\omega_{a}}{\delta_{w}}=\frac{2\gamma_{\mathrm{lg}}\left(1+\cos\theta_{Y}\right)}{\delta_{w}}$$

Under subzero conditions, a quasi-liquid layer or unfrozen water may still exist at the ice–coating interface. Therefore, the interfacial interaction between water and the coating surface can, to some extent, reflect the adhesion behavior between ice and the coating surface [34]. If a correlation coefficient α is assumed, it can be determined from the ratio $F_{\mathrm{Ice}}/F_{w}$ as follows:

(10) $$\alpha =\frac{F_{\mathrm{Ice}}}{F_{w}}=\frac{\varepsilon_{w}\delta_{w}^{2}}{\varepsilon_{\mathrm{Ice}}\delta_{\mathrm{Ice}}^{2}}$$

where $F_{\mathrm{Ice}}$ is the electrostatic interaction force between ice and the coating surface; $\varepsilon_{\mathrm{Ice}}$ is the relative permittivity of ice; and $\delta_{\mathrm{Ice}}$ is the equivalent distance between the ice molecules and the surface molecules of the coating.

Ice adhesion strength $P_{\mathrm{Ice}}$ on the coating surface:

(11) $$P_{\mathrm{Ice}}=\frac{F_{\mathrm{Ice}}}{S_{a}}=\frac{\alpha F_{w}}{S_{a}}=\frac{2\gamma_{\mathrm{lg}}\left(1+\cos\theta_{Y}\right)\varepsilon_{w}\delta_{w}}{\varepsilon_{\mathrm{Ice}}\delta_{\mathrm{Ice}}^{2}}$$

**Figure 16:**
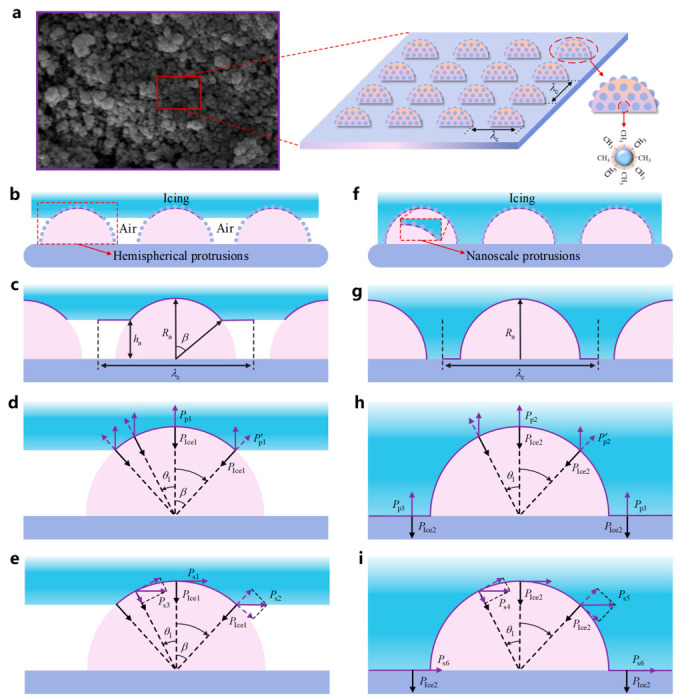
Schematic illustration of the theoretical analysis of ice adhesion strength on the superhydrophobic coating surface. (**a**) Schematic of the simplified microstructural model; (**b**) Cassie–Baxter state model; (**c**) calculation model for the Cassie–Baxter state; (**d**) calculation model for ice peel strength in the Cassie–Baxter state; (**e**) calculation model for ice shear strength in the Cassie–Baxter state; (**f**) Wenzel state model; (**g**) calculation model for the Wenzel state; (**h**) calculation model for ice peel strength in the Wenzel state; (**i**) calculation model for ice shear strength in the Wenzel state.

The derivation of Equation (11) is based on an ideal smooth coating surface assumed in the Young equation. As shown in Figure 16a, the surface of the superhydrophobic coating exhibits micro/nanoscale rough structures with different sizes and geometrical characteristics. To compensate for the effect of coating roughness on the contact angle, the surface morphology of the superhydrophobic coating was abstracted and simplified. It was assumed that hemispherical protrusions with identical shape and size were uniformly distributed on the coating surface, with a spacing of $\lambda_{c}$ between adjacent protrusions, and that each protrusion was further covered with a large number of nanoscale protrusions. According to the derivation of ice adhesion strength by Guy Fortin et al. [35] based on differences in contact mechanisms, Equation (11) was modified as follows:

(12) $$P_{\mathrm{Ice}}=\frac{2\gamma_{\mathrm{lg}}\left(1+\cos\theta_{Y}\right)\varepsilon_{w}\delta_{w}\tau }{\varepsilon_{\mathrm{Ice}}\delta_{\mathrm{Ice}}^{2}}$$

(13) $$\tau =\frac{\delta_{\mathrm{Ice}}}{R_{q}}+\left(1-\frac{\delta_{\mathrm{Ice}}}{R_{q}}\right)f_{\mathrm{Ia}}$$

where τ is the correction factor accounting for the effect of coating surface roughness on the static contact angle; $f_{\mathrm{Ia}}$ is the ratio of the actual ice–coating contact area to the apparent contact area; and $R_{q}$ is the roughness of the coating surface.

### 3.2. Model Construction in the Cassie–Baxter State

When a water droplet begins to freeze on the surface of the superhydrophobic coating, its contact angle remains above 150° under the influence of the hierarchical micro/nanostructure, resulting in a typical Cassie–Baxter contact state between the ice and the coating, as shown in Figure 16b. In this state, the ice layer does not fully wet the coating surface, but is instead supported by the trapped air layer between adjacent hemispherical protrusions. The continuous air layer retained within the micro/nanostructures not only significantly reduces the actual contact area between the ice layer and the coating, but also increases the interfacial thermal resistance by hindering interfacial heat transfer.

According to the adhesion behavior between ice and the surface of the superhydrophobic coating in the Cassie–Baxter contact state, although some air also exists between the nanoscale protrusions distributed on the hemispherical protrusions and the ice, the actual contact area accounts for only a very small fraction of the surface area of the protrusion structures because the static contact angle is greater than 150°. Therefore, the nanoscale protrusions were neglected, and the model was established as shown in Figure 16c.

In this study, the spacing $\lambda_{c}$ between adjacent hemispherical protrusions was taken as one basic ice adhesion unit. Within this unit, under the Cassie–Baxter contact state, the actual contact area of ice is the curved surface at the top of the hemispherical protrusion, while the apparent contact area corresponds to the region indicated by the purple line in Figure 16c. It was assumed that the ice layer at the contact interface between the ice and the protrusions was horizontal and had a uniform height. Here, $h_{a}$ is the height of the air gap, $R_{a}$ is the radius of the protrusion, and β is half of the central angle corresponding to the actual contact area of the ice. Accordingly, under the Cassie–Baxter contact state, the adhesion strength $P_{\mathrm{Ice}1}$ of ice on the superhydrophobic coating surface can be expressed as the weighted sum of the adhesion strengths of the contact and non-contact regions:

(14) $$P_{\mathrm{Ice}1}=\frac{2\gamma_{\mathrm{lg}}\left(1+\cos\theta_{Y}\right)\varepsilon_{w}\delta_{w}\tau f_{\mathrm{Ia}}}{\varepsilon_{\mathrm{Ice}}\delta_{\mathrm{Ice}}^{2}}+\frac{2\gamma_{\mathrm{lg}}\left(1+\cos\theta_{s}\right)\varepsilon_{s}\delta_{s}\tau \left(1-f_{\mathrm{Ia}}\right)}{\varepsilon_{\mathrm{Ice}}\delta_{\mathrm{Ice}}^{2}}$$

where $\theta_{s}$ is the static contact angle of the water droplet in air, with $\theta_{s}=$180°; $\varepsilon_{s}$ is the relative permittivity of air; and $\delta_{s}$ is the equivalent distance between the frozen-state water molecules and the air molecules over the apparent contact area.

Within one basic ice adhesion unit, the length of the actual contact area between the protrusion and the ice is denoted by $L_{1}$:

(15) $$L_{1}=2\beta R_{a}$$

where $\beta =\arccos\left(h_{a}/R_{a}\right)$.

The contact length between the ice and the air layer is denoted by $L_{2}$:

(16) $$L_{2}=\lambda_{c}-2R_{a}\sin\beta$$

The contact length corresponding to the apparent contact area of the ice is denoted by L:

(17) $$L=2\beta R_{a}+\lambda_{c}-2R_{a}\sin\beta$$

According to the definition of $f_{\mathrm{Ia}}$, it can be obtained that:

(18) $$f_{\mathrm{Ia}}=\frac{L_{1}}{L}=\frac{2\beta R_{a}}{\lambda_{c}+2R_{a}\left(\beta -\sin\beta \right)}$$

By combining Equations (14) and (18), the adhesion strength $P_{\mathrm{Ice}1}$ within each basic ice adhesion unit under the Cassie–Baxter contact state can be obtained as follows:

(19) $$P_{\mathrm{Ice}1}=\frac{4\beta R_{a}\gamma_{\mathrm{lg}}\left(1+\cos\theta_{Y}\right)\varepsilon_{w}\delta_{w}\tau }{\varepsilon_{\mathrm{Ice}}\delta_{\mathrm{Ice}}^{2}\left(\lambda_{c}+2R_{a}\left(\beta -\sin\beta \right)\right)}$$

Ice peel strength and ice shear strength are important parameters for evaluating the anti-icing performance of materials, and they also reflect the ice adhesion under different loading directions. Based on the adhesion strength within the basic ice adhesion unit under each contact state, quantitative analysis was carried out using a numerical calculation method. Under the Cassie–Baxter contact state, the force distribution corresponding to the ice peel strength over the actual contact area is shown in Figure 16d, where the arrows indicate the directions of the forces associated with the pressure. At any point on the curved contact surface between the ice and the protrusion, the component of the peel strength $P_{p1}$ along the line connecting the contact point to the circle center, ${P^{\prime }}_{p1}$, is in equilibrium with the adhesion strength $P_{\mathrm{Ice}1}$.

Based on the force analysis, the peel strength $P_{p1}$ is given by:

(20) $$P_{p1}=\frac{P_{\mathrm{Ice}1}}{\cos\theta_{1}}$$

where $\theta_{1}$ is the angle between the line from an arbitrary contact point to the circle center and the vertical line through the circle center.

Within one basic ice adhesion unit, the average peel strength $\bar{P_{\mathrm{pa}}}$ is:

(21) $$\bar{P_{\mathrm{pa}}}=\frac{\int_{0}^{\beta }P_{p1}d\theta_{1}}{\beta }=\frac{P_{\mathrm{Ice}1}\ln\left(\sec\beta +\tan\beta \right)}{\beta }$$

Under the Cassie–Baxter contact state, the force distribution corresponding to the ice shear strength over the actual contact area is shown in Figure 16e. When the protrusion is subjected to an external horizontal force toward the right, and assuming that the protrusion structure remains intact, the ice in the middle, left, and right regions experiences frictional forces from the protrusion surface. The components of the shear strength along the surface direction of the protrusion are in equilibrium with these frictional forces.

Based on the force analysis at each point, the shear strength on the surface of the protrusion can be expressed as follows:

(22) $$P_{s1}=\mu P_{\mathrm{Ice}1}$$

(23) $$P_{s2}=\frac{\mu P_{\mathrm{Ice}1}}{\cos\theta_{1}}$$

(24) $$P_{s3}=\frac{\mu P_{\mathrm{Ice}1}}{\cos\theta_{1}-\mu \sin\theta_{1}}$$

where μ is the friction coefficient of the superhydrophobic coating surface; $P_{s1}$, $P_{s2}$ and $P_{s3}$ denote the shear strengths in the middle, right, and left regions of the protrusion structure, respectively; and when $\theta_{1}=$ 0°, $P_{s1}=P_{s2}=P_{s3}$.

According to the pressure balance relationship, the average value can be obtained by integration as follows:

(25) $$\bar{P_{s2}}=\frac{\int_{0}^{\beta }P_{s2}d\theta_{1}}{\beta }=\frac{\mu P_{\mathrm{Ice}1}\ln\left(\sec\beta +\tan\beta \right)}{\beta }$$

(26) $$\bar{P_{s3}}=\frac{\int_{0}^{\beta }P_{s3}d\theta_{1}}{\beta }=\frac{\mu P_{\mathrm{Ice}1}}{\beta \sqrt{1+\mu^{2}}}\ln\left(\frac{\sec\left(\beta +\phi \right)+\tan\left(\beta +\phi \right)}{\sec\phi +\tan\phi }\right)$$

where ϕ=arctanμ.

Within one basic ice adhesion unit, the average shear strength $\bar{P_{\mathrm{sa}}}$ is:

(27) $$\bar{P_{\mathrm{sa}}}=\left(\bar{P_{s2}}+\bar{P_{s3}}\right)/2$$

### 3.3. Model Construction in the Wenzel State

Under low-temperature and high-humidity conditions, water molecules can readily infiltrate the micro/nanoporous structures on the superhydrophobic coating surface and condense therein, causing the solid–liquid interface containing an air layer (Cassie–Baxter state) to transition into the fully wetted Wenzel state. In general, the actual contact ratio between ice and the substrate on a superhydrophobic surface is typically less than 10% [36]. Combined with the observed experimental results, during the gradual freezing of water on the superhydrophobic coating surface, some droplets may expand upon freezing or be disturbed by external factors such as vibration, pressure fluctuations, or dynamic bouncing of other droplets. As a result, the air beneath the droplets is gradually expelled, causing the droplets to partially or completely contact the superhydrophobic surface and thereby transition into the Wenzel wetting state, as shown in Figure 16f.

To further analyze the adhesion behavior between ice and the superhydrophobic coating surface in the Wenzel wetting state, the principal difference between the Wenzel state and the Cassie–Baxter state lies in whether an air layer exists within the micro/nanostructure gaps. After neglecting the nanoscale protrusions, the model was established as shown in Figure 16g.

Within one basic ice adhesion unit, the ice is in complete contact with the coating surface, and $f_{\mathrm{Ia}}=1$. Therefore, the adhesion strength $P_{\mathrm{Ice}2}$ between the ice and the superhydrophobic coating surface in the Wenzel wetting state can be expressed as:

(28) $$P_{\mathrm{Ice}2}=\frac{2\gamma_{\mathrm{lg}}\left(1+\cos\theta_{Y}\right)\varepsilon_{w}\delta_{w}\tau }{\varepsilon_{\mathrm{Ice}}\delta_{\mathrm{Ice}}^{2}}$$

The length of the actual contact area between the protrusion and the ice is denoted by ${L^{\prime }}_{1}$:

(29) $${L^{\prime }}_{1}=\pi R_{a}$$

The contact length between the base surface and the ice is denoted by ${L^{\prime }}_{2}$:

(30) $${L^{\prime }}_{2}=\lambda_{c}-2R_{a}$$

The contact length corresponding to the apparent contact area of the ice is denoted by $L^{\prime }$:

(31) $$L^{\prime }=\pi R_{a}+\lambda_{c}-2R_{a}$$

Under the Wenzel wetting state, the force distribution corresponding to the ice peel strength over the actual contact area is shown in Figure 16h. According to the equilibrium relation at each point, the expressions for the peel strength on the protrusion surface and the base surface are given as follows:

(32) $$P_{p2}=\frac{P_{\mathrm{Ice}2}}{\cos\theta_{1}}$$

(33) $$P_{p3}=P_{\mathrm{Ice}2}$$

According to the pressure balance relationship, the average value can be obtained by integration as follows:

(34) $$\bar{P_{p2}}=\frac{2\int_{0}^{\pi /2}P_{p2}d\theta_{1}}{\pi }\approx 3P_{\mathrm{Ice}2}$$

(35) $$\bar{P_{p3}}=P_{\mathrm{Ice}2}$$

The average peel strength in the Wenzel state is $\bar{P_{\mathrm{pb}}}$:

(36) $$\bar{P_{\mathrm{pb}}}=\frac{3P_{\mathrm{Ice}2}\pi R_{a}+\left(\lambda_{c}-2R_{a}\right)P_{\mathrm{Ice}2}}{\pi R_{a}+\lambda_{c}-2R_{a}}$$

The force distribution corresponding to the ice shear strength over the actual contact area is shown in Figure 16i. The expressions for the shear strength on the protrusion structure and the base surface are given as follows:

(37) $$P_{s4}=\frac{\mu P_{\mathrm{Ice}2}}{\cos\theta_{1}-\mu \sin\theta_{1}}$$

(38) $$P_{s5}=\frac{\mu P_{\mathrm{Ice}2}}{\cos\theta_{1}}$$

(39) $$P_{s6}=\mu P_{\mathrm{Ice}2}$$

where $P_{s4}$ and $P_{s5}$ are the shear strengths in the left and right regions of the protrusion structure, respectively; and $P_{s6}$ is the shear strength on the base surface.

The average value can be obtained after integration as follows:

(40) $$\bar{P_{s4}}=\frac{2\mu P_{\mathrm{Ice}2}\int_{0}^{\pi /2}P_{s4}d\theta_{1}}{\pi }$$

(41) $$\bar{P_{s5}}\approx 3\mu P_{\mathrm{Ice}2}$$

(42) $$\bar{P_{s6}}=\mu P_{\mathrm{Ice}2}$$

The average shear strength in the Wenzel state is Wenzel $\bar{P_{\mathrm{sb}}}$:

(43) $$\bar{P_{\mathrm{sb}}}=\frac{\pi R_{a}\left(\bar{P_{s4}}+\bar{P_{s5}}\right)+2\left(\lambda_{c}-2R_{a}\right)\bar{P_{s6}}}{\pi R_{a}+2\left(\lambda_{c}-2R_{a}\right)}$$

**Figure 17:**
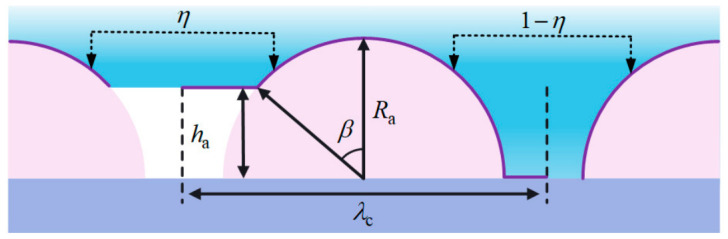
Calculation model for the mixed Cassie–Baxter and Wenzel states.

As shown in Figure 17, assuming that within a given area the proportion of ice in the Cassie–Baxter contact state is η and that in the Wenzel wetting state is 1−η, the ice peel strength $P_{p}$ and ice shear strength $P_{s}$ between the ice and the coating surface can be obtained from the above derivations as follows:

(44) $$P_{p}=\eta \bar{P_{\mathrm{pa}}}+\left(1-\eta \right)\bar{P_{\mathrm{pb}}}$$

(45) $$P_{s}=\eta \bar{P_{\mathrm{sa}}}+\left(1-\eta \right)\bar{P_{\mathrm{sb}}}$$

### 3.4. Analysis of Factors Affecting Ice Adhesion Strength

Based on the experimental results and relevant literature, the calculation parameters of the superhydrophobic coating used in this study were determined, as listed in Table 3.

By substituting the parameters in Table 3 into the Cassie–Baxter contact state model, the calculated ice peel strength and ice shear strength were 23.41 kPa and 6.58 kPa, respectively. Comparison between the experimental and theoretical results of ice adhesion strength shows that the theoretical values were generally higher than the measured values, indicating that a certain deviation still exists between the theoretical model and the actual surface structure. This discrepancy may be attributed to the following reasons. First, the influence of the nanoscale protrusions on the protrusion surface was not considered in the modeling process, whereas the tiny air gaps formed between these nanostructures and the ice can further weaken the interfacial contact and adhesion; Second, the protrusion structures were idealized as hemispherical in the model, whereas the actual protrusions exhibit obvious differences in morphology, size, and distribution density, making the real surface more complex than the theoretical model. Because the above simplified assumptions could not fully reflect the actual microstructural characteristics of the coating surface, the theoretical results were somewhat higher than the measured values. However, since the relative errors remained within a reasonably small range, the theoretical models established in this study for ice peel strength and ice shear strength are considered to have good accuracy.

**Figure 18:**
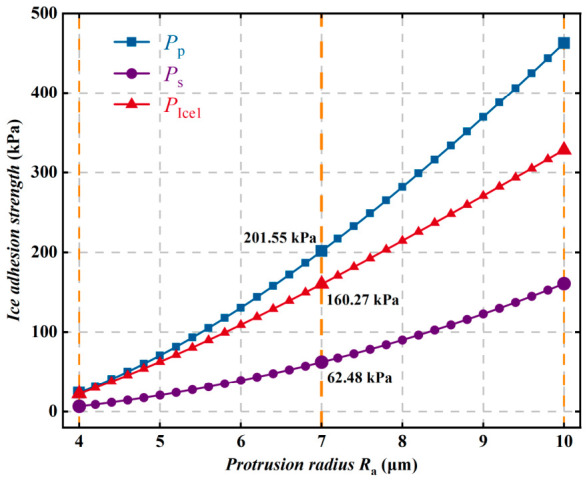
Variation in ice adhesion strength with protrusion radius $R_{a}$.

**Figure 19:**
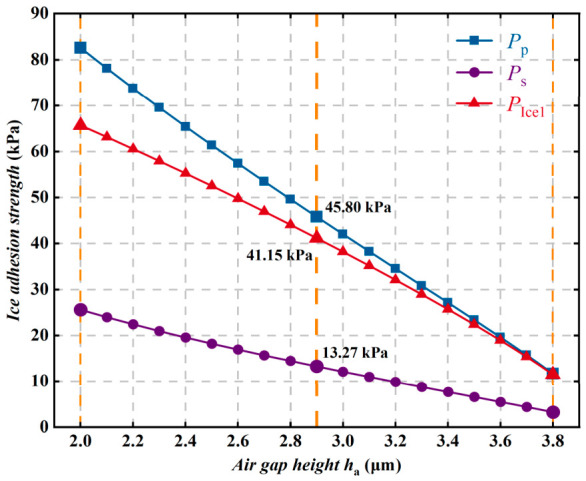
Variation in ice adhesion strength with air gap height $h_{a}$.

**Figure 20:**
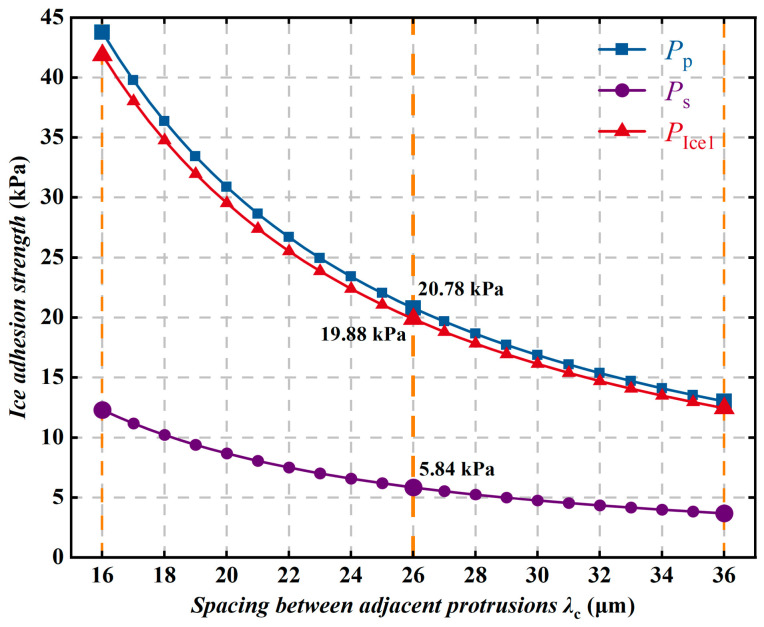
Variation in ice adhesion strength with the spacing $\lambda_{c}$ between adjacent protrusions.

**Figure 21:**
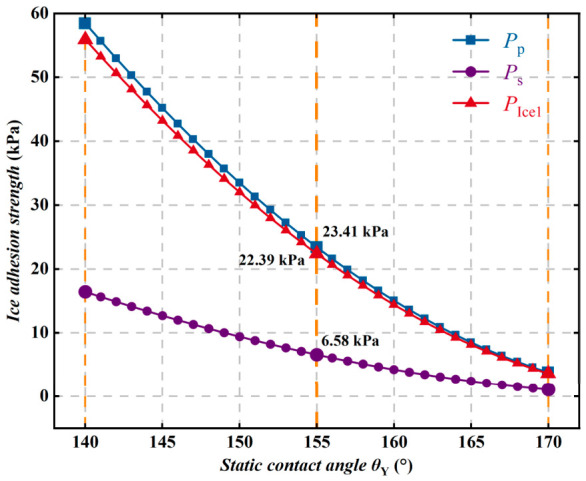
Variation in ice adhesion strength with static contact angle $\theta_{Y}$.

**Figure 22:**
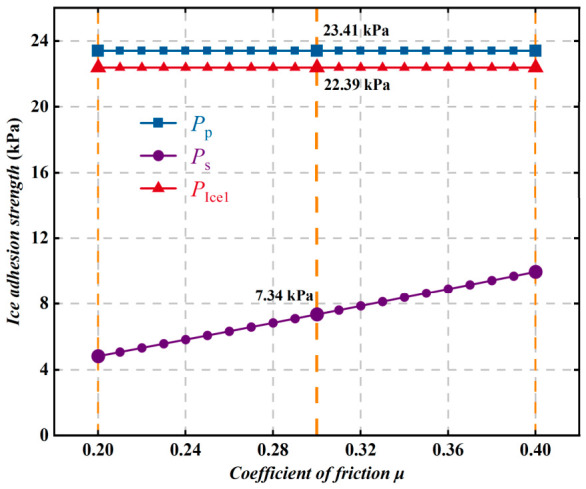
Variation in ice adhesion strength with coefficient of friction μ.

When the superhydrophobic coating and ice are in the Cassie–Baxter contact state, the variations in ice adhesion strength with model parameters are shown in Figure 18, Figure 19, Figure 20, Figure 21 and Figure 22. As shown in Figure 18, with the gradual increase in the protrusion radius $R_{a}$, the ice adhesion strength, ice peel strength, and ice shear strength all exhibit a significant monotonic increasing trend. Among them, the ice peel strength is consistently higher than the ice shear strength. As $R_{a}$ increases from 7 μm to 10 μm, the ice peel strength and ice shear strength increase by 261.12 kPa and 98.37 kPa, respectively, and the rate of increase in ice peel strength is also significantly greater than that in ice shear strength. These results indicate that the protrusion radius on the superhydrophobic coating surface has a more significant influence on ice peel strength. Further analysis shows that increasing the protrusion radius enlarges the effective contact area and reduces the retention of the interfacial air layer, thereby strengthening intermolecular interactions. However, excessively large microstructures may lead to excessively close interfacial contact, which in turn greatly increases the difficulty of deicing.

As shown in Figure 19, when $h_{a}$ increases from 2.0 μm to 3.8 μm, the ice peel strength decreases from 82.6 kPa to about 11.7 kPa, corresponding to a reduction of as much as 85.5%, whereas the decrease in ice shear strength is relatively moderate. Meanwhile, when $h_{a}$ increases to 3.6 μm, the difference between the adhesion strength and the ice peel strength decreases markedly and tends to become negligible. Further analysis indicates that, as the air gap height increases, the physical separation between the ice and the coating substrate becomes larger, resulting in a significant reduction in the effective solid–liquid contact area and thereby weakening the intermolecular interactions at the interface. In addition, a larger air gap effectively reduces the interfacial fracture energy and induces stress concentration at the tops of the microstructures, making the ice more likely to detach from the surface.

As shown in Figure 20, the ice peel strength and ice shear strength decrease nonlinearly with increasing spacing $\lambda_{c}$ between adjacent protrusions, with the ice shear strength remaining significantly lower. Meanwhile, the ice adhesion strength and ice peel strength vary synchronously. The variation in ice shear strength is relatively gradual, whereas when $\lambda_{c}$ increases from 16 μm to 26 μm, the ice peel strength decreases by 22.98 kPa, which is clearly greater than the corresponding change in ice shear strength. The spacing between adjacent protrusions on the superhydrophobic coating surface reflects the degree of dispersion of the rough structures. The calculated results show that a larger value of $\lambda_{c}$ corresponds to better anti-icing performance of the coating. However, according to relevant experimental studies [38,39], when $\lambda_{c}$ exceeds a certain value, the rough structures on the superhydrophobic coating surface become too sparse to maintain hydrophobicity, and the ice adhesion strength then increases. Further analysis indicates that an increase in $\lambda_{c}$ means a reduction in the number of protrusions per unit area, which decreases the proportion of the coating top surface that can directly contact the ice and thus reduces the stress that can be transmitted across the interface. Excessively large gaps may reduce the mechanical stability and abrasion resistance of the coating, and droplets may also more readily penetrate into the microstructural gaps under gravity, resulting in an increase in ice adhesion strength. Therefore, only an appropriate distribution density of rough structures can effectively reduce ice adhesion strength.

As the static contact angle $\theta_{Y}$ gradually increases, the ice adhesion strength of the superhydrophobic coating decreases significantly, indicating that the stronger the hydrophobicity of the coating, the lower the ice adhesion strength, as shown in Figure 21. Within the tested contact angle range of 140° to 150°, the ice peel strength and adhesion strength remain at relatively high levels but decrease synchronously, whereas the ice shear strength is consistently much lower than the other two. When the contact angle further increases to 170°, the ice adhesion strength decreases to approximately 2~4 kPa, indicating that both the actual interfacial contact area and the interfacial energy are significantly reduced. These results demonstrate that improving surface hydrophobicity can effectively reduce ice adhesion strength and thereby markedly enhance the anti-icing performance of the material.

As shown in Figure 22, the coefficient of friction μ mainly determines the shear failure behavior at the ice–coating interface during loading, while its influence on peel failure and the intrinsic interfacial adhesion is relatively limited. As the surface friction coefficient decreases, the tendency of the ice layer to slide along the interface becomes stronger, and the ability of the interface to transmit shear loads is weakened, making shear failure more likely to occur and ultimately leading to a reduction in ice adhesion strength. Therefore, in the structural design and surface regulation of superhydrophobic anti-icing materials, the optimization of surface friction characteristics should be emphasized. By constructing low-friction surfaces, the shear adhesion strength of ice can be effectively reduced, thereby further improving the anti-icing and deicing performance of the material.

**Figure 23:**
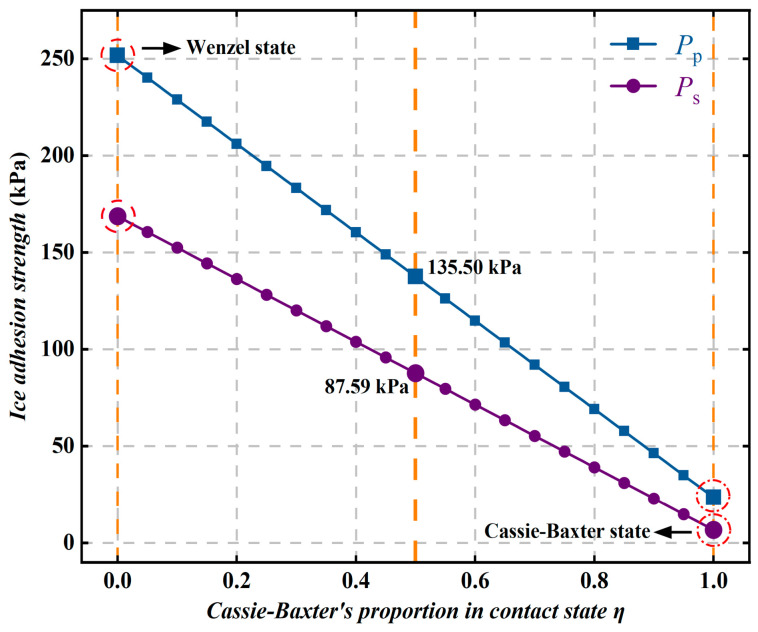
Variation in ice adhesion strength with the proportion of the Cassie–Baxter contact state η.

When the ice and the coating surface are in a mixed contact state, the interface is generally composed of a fully wetted solid–liquid contact region and an air-gap region. As shown in Figure 23, as the proportion of the Cassie–Baxter contact state η increases from 0 to 1, both the ice peel strength and the ice shear strength of the superhydrophobic coating decrease markedly. Specifically, the ice peel strength decreases from 251.59 kPa to 23.41 kPa, and the ice shear strength decreases from 168.60 kPa to 6.58 kPa, corresponding to reductions of approximately 90.7% and 96.1%, respectively. These results indicate that the Cassie–Baxter state can significantly reduce the actual contact area and interfacial adhesion between ice and the solid surface, thereby markedly lowering the ice adhesion strength. This difference mainly arises from the significantly increased interfacial contact area in the Wenzel wetting state. In this state, the ice not only contacts the tops of the rough structures, but also comes into contact with the relatively smooth base surface, thereby enhancing interfacial adhesion. At the same time, mechanical interlocking may form between the surface microstructures and the ice layer, so that interfacial failure requires overcoming not only the adhesion at the ice–solid interface, but also part of the cohesive strength of the ice itself. Compared with the Wenzel state, the ice layer in the Cassie–Baxter state is more prone to sliding and peeling. To some extent, the presence of the air layer can hinder the downward movement of the contact line under the effects of gravity, Laplace pressure difference, and external disturbances, thereby reducing the transition of the ice layer to the Wenzel wetting state. Therefore, maintaining a stable Cassie–Baxter contact state is the key to improving the anti-icing performance of superhydrophobic coatings.

## 4. Conclusions

In this study, an abrasion-resistant layered superhydrophobic coating composed of a primer and a topcoat was fabricated. Through performance testing, theoretical analysis, and numerical calculation, the accuracy of the theoretical model for ice adhesion strength was verified, and the effects of surface micro/nanostructure, wetting characteristics, interfacial friction properties, and contact mode on the anti-icing performance of the coating were systematically revealed.

(1) The coating exhibited robust durability and low ice adhesion, maintaining a static contact angle of 146.5° even after 90 sand impacts. The measured ice peel and shear strengths were 22.36 kPa and 5.87 kPa, respectively, with small data fluctuations.

(2) Surface geometry and physical properties significantly affect ice adhesion. While optimizing protrusion spacing and air gap height helps reduce adhesion strength, excessively large spacing may compromise structural stability. Additionally, higher static contact angles significantly reduce ice adhesion, while the coefficient of friction mainly influences the interfacial shear failure behavior.

(3) The contact state at the ice–coating interface plays a decisive role in anti-icing performance. As the proportion of the Cassie–Baxter contact state increases, the ice adhesion strength decreases significantly. If the interface transitions to the Wenzel wetting state, the increase in actual interfacial contact area and the potential mechanical interlocking effect will lead to a marked increase in ice adhesion strength. Therefore, maintaining a stable Cassie–Baxter contact state is the key to improving the anti-icing performance of superhydrophobic coatings.

Future work should focus on refining the theoretical model by incorporating complex interfacial interactions, such as van der Waals forces and mechanical interlocking, while accounting for the effects of ambient temperature and humidity on dynamic wetting-state transitions. Furthermore, comprehensive evaluations of insulation performance and long-term aging are essential to fully assess the coating’s reliability and application potential in practical high-voltage insulation and anti-icing environments.

## Figures and Tables

**Table 1:** Raw materials required for coating preparation.

No.	Raw Material	Specification	Manufacturer (Co., Ltd.)
1	bisphenol A epoxy resin	175~185 g/mol	Sinopharm Chemical Reagent, Shanghai, China (ShC)
2	amino silicone oil	SD-815	Guangzhou Xinguan Chemical Technology, Guangzhou, China
3	acetone	AR	Sinopharm Chemical Reagent (ShC)
4	anhydrous ethanol	AR, content ≥ 99.7%	Sinopharm Chemical Reagent (ShC)
5	aqueous ammonia	AR, content 25%	Sinopharm Chemical Reagent (ShC)
6	TEOS	AR	Sinopharm Chemical Reagent (ShC)
7	HMDS	AR, water content < 100 ppm	Keyisheng New Materials, Jinan, China
8	silane coupling agent	KH560	Kangjin New Materials Technology, Dongguan, China
9	nylon powder	PA11, 500 mesh	Shanghai Aiyu Plastics, Shanghai, China

**Table 2:** Equipment used for coating preparation and testing.

No.	Equipment	Model	Manufacturer (Co., Ltd.)
1	precision electronic balance	JA5003	Shanghai LNB Instrument, Shanghai, China
2	low-pressure high-atomization spray gun	LPH-101-LVG	Guanpin Machinery and Equipment, Shenzhen, China
3	magnetic stirrer	ZNCL-BS	Shanghai LNB Instrument, Shanghai, China
4	low-temperature test chamber	DW-50	Kaijian Precision Instrument, Guangzhou, China
5	benchtop forced-air drying oven	DH-420	Marit Technology, Wuxi, China
6	contact angle meter	KZS-30	Kezhong Precision Instrument, Dongguan, China
7	digital push-pull force gauge	DS2-1000N	Xihao Industry, Shanghai, China
8	FE-SEM	Nova NanoSEM 450	Thermo Fisher Scientific, Waltham, MA, USA
9	X-ray photoelectron spectrometer	AXIS SUPRA+	Kratos Analytical Ltd., Manchester, UK
10	ultrasonic cleaner	UCM-6K	Shanghai LNB Instrument, Shanghai, China

**Table 3:** Theoretical calculation parameters.

Symbol	Parameter	Value
$\varepsilon_{w}$	relative permittivity of water	78
$\varepsilon_{\mathrm{Ice}}$	relative permittivity of ice	2.8
$\delta_{w}$	distance between water and coating surface molecules	0.7 μm [35]
$\delta_{\mathrm{Ice}}$	distance between ice and coating surface molecules	0.75 μm [35]
$\gamma_{\mathrm{lg}}$	surface tension at the liquid–gas interface	7.26 × 10^−2^ ${N/m}^{2}$ [37]
$\theta_{Y}$	static contact angle	155°
$\lambda_{c}$	spacing between adjacent protrusions	24 μm
$R_{q}$	surface roughness of the coating	5.36 μm
$h_{a}$	height of the air gap	3.5 μm
$R_{a}$	radius of the protrusion	4 μm
μ	friction coefficient of the coating surface	0.27

## Data Availability

Data will be made available on request.
